# IL-10 plus the EASIX score predict bleeding events after anti-CD19 CAR T-cell therapy

**DOI:** 10.1007/s00277-023-05477-y

**Published:** 2023-10-09

**Authors:** Xindi Wang, Chenggong Li, Wenjing Luo, Yinqiang Zhang, Zhongpei Huang, Jia Xu, Heng Mei, Yu Hu

**Affiliations:** 1 Institute of Hematology, Union Hospital, Tongji Medical College, Huazhong University of Science and Technology, Wuhan, 430022 China; 2 Hubei Clinical Medical Center of Cell Therapy for Neoplastic Disease, Wuhan, 430022 China

**Keywords:** Chimeric antigen receptor T-cell therapy, Bleeding events, Endothelial activation and stress index, Interleukin-10

## Abstract

Chimeric antigen receptor (CAR) T-cell-associated coagulopathy can cause bleeding events. To explore risk factors for hemorrhage after CAR T-cell therapy, we retrospectively analyzed routine indicators in 56 patients with non-Hodgkin lymphoma and B-cell acute lymphoblastic leukemia who received anti-CD19 CAR T-cell therapy. Disturbance of coagulation occurred mainly within one month post infusion, especially on day 7 and 14. The cumulative incidence of bleeding events within one month was 32.8%, with the median onset of 7 (range, 0–28) days. All bleeding events were grade 1–3. Patients who experienced bleeding events within one month had longer prothrombin time, higher IL-6, higher IL-10, and lower platelets before lymphodepletion. There were also correlations among coagulation-, inflammatory-, and tumor burden-related markers. Multi-variate analysis showed IL-10 (> 7.98 pg/mL; adjusted odds ratio [OR], 13.84; 95% confidence interval [CI], 2.03–94.36; *P* = 0.007) and the endothelial activation and stress index (EASIX, defined as dehydrogenase [U/L] × creatinine [mg/dL] / platelets [×10^9^ cells/L]; >7.65; adjusted OR, 7.06; 95% CI, 1.03–48.23; *P* = 0.046) were significant risk factors for bleeding events. IL-10 plus the EASIX defined three risk groups for bleeding events with cumulative incidence of 100% (hazard ratio [HR], 14.47; 95% CI, 2.78–75.29; *P* < 0.0001), 38.5% (HR, 3.68; 95% CI, 0.82–16.67; *P* = 0.089), and 11.8% (reference), respectively. Future studies are needed to verify the risk assessment models for bleeding events after CAR T-cell treatment in larger cohorts.

**Supplementary Information:**

The online version contains supplementary material available at 10.1007/s00277-023-05477-y.

## Introduction

Chimeric antigen receptor (CAR) T-cell therapy is a revolutionary treatment option and has achieved breakthroughs in the field of hematological oncology. In particular, CD19-targeted CAR T-cell therapy has achieved remarkable and durable efficacy in refractory/relapsed B-cell malignancies [1–3]. Currently, four CD19-targeted products have been approved by the Food and Drug Administration, including tisagenlecleucel (tisa-cel), axicabtagene ciloleucel (axi-cel), brexucabtagene autoleucel (brexu-cel), and lisocabtagene maraleucel (liso-cel). Additionally, many novel CAR T-cells are undergoing clinical trials.

CAR T-cell treatment also causes toxicities, most typically cytokine release syndrome (CRS) and immune-effector cell-associated neurotoxicity syndrome (ICANS). However, treatment-related coagulopathy is also a major and frequent adverse event. CAR T-cell-associated coagulopathy (CARAC) is a clinical syndrome characterized by bleeding and/or thrombosis accompanied by decreased platelets (PLTs) and coagulopathy, which occurs mainly within 28 days after CAR-T cell infusion [4]. During CAR T-cell treatment, more than 50% of patients will experience thrombocytopenia or have at least one coagulation abnormality [5–10]. Severe treatment-related coagulopathy may be fatal [11–13]. Bleeding events occur in about 19.6% of patients during CAR T-cell treatment [4], but the current disseminated intravascular coagulation scoring system is less suitable for predicting these events [13]. The frequency of thrombotic events during CAR T-cell treatment ranged from 6.9 to 11%, and the low incidence limits further statistical analysis [13, 14]. Studies have shown that PLTs before lymphodepleting chemotherapy [13], CRS [8, 10, 15], ICANS [13], and tumor necrosis factor α (TNFα) [16] are associated with bleeding events. Generally, interactions exist between the coagulation system, inflammation, and tumors [13, 17–19]. However, current risk prediction models lack coverage of all three aspects. Therefore, a comprehensive and convenient predictive model for bleeding events during CAR T-cell treatment is needed.

The endothelium is important for maintaining homeostasis in the coagulation system. Its integrity contributes to maintain anticoagulant properties and control of platelet adhesion and activation [20]. In addition, endothelial cells partially regulate blood vessels in response to bleeding events [20]. High lactic dehydrogenase (LDH), high creatinine, and low platelets are important indicators of thrombotic microangiopathy after allogeneic transplantation [21]. The endothelial activation and stress index (EASIX) score, defined as LDH (U/L) × creatinine (mg/dL) / PLTs (×10^9^/L), is used to predict endothelial complications and mortality in allogeneic transplantation and acute graft-versus-host disease [22]. There are also modified EASIX variants to predict CRS and ICANS during CAR T-cell treatment [23–25]. Because endothelial activation triggers fluctuations in coagulation, we speculate that the EASIX score could also be used to predict and guide management of hemorrhage caused by coagulopathy during CAR T-cell therapy.

In this retrospective study, we analyzed fluctuations in coagulation, recorded bleeding episodes, as well as screened coagulation-, inflammation-, and tumor burden-related indicators for risk stratification of bleeding events during CAR T-cell treatment in a single-center cohort of 56 patients.

## Methods

### Patient and clinical data collection

We conducted a retrospective analysis of patients with hematological malignances including non-Hodgkin lymphoma (NHL) and B-cell acute lymphoblastic leukemia (B-ALL) treated with anti-CD19 CAR T-cells from a clinical trial (NCT04008251). The production and treatment strategies of CAR T-cells in the clinical trial are consistent with previous studies [26]. The second-generation CAR structure used in the study contains a humanized CD19 single chain variable fragment, CD137 costimulatory domain, and CD3ζ signal sequence [27]. Patients in the study received lymphodepleting chemotherapy (fludarabine 30mg/m^2^ and cyclophosphamide 250mg/m^2^) on day (D) -5– -3, followed by infusion of CAR T-cells at 1×10^6^ cells/kg on D0 and D1, respectively. Fifty-six patients successfully received infusion and follow-up visits. Patients in the clinical trial signed informed consent consistent with the Declaration of Helsinki.

We collected routine coagulation-related indicators including PLTs, D-dimer, prothrombin time (PT), activated partial thromboplastin time (APTT), thrombin time (TT), fibrinogen (FIB), fibrinogen degradation products (FDP), international normalized ratio (INR), antithrombin III (ATIII); inflammatory markers, including C-reactive protein (CRP), ferritin, interleukin (IL)-2, IL-4, IL-6, IL-10, interferon (IFN) γ, TNFα; tumor burden-related indicators, including LDH and bone-marrow blast. Recorded timepoints included pre-lymphodepletion (pre-LD), days 0, 7, 14, months (M) 1, 3, 6, and 9 after infusion. In addition, we recorded the peak and minimum values of these indicators within one month.

### Grading of the coagulation parameters

The grading of thrombocytopenia, hypofibrinogenemia, increased INR, and prolonged APTT was according to Common Terminology Criteria for Adverse Events, version 5.0. The rating assessment of D-dimer, TT, PT, ATIII, and FDP refers to the classification as previously used (supplemental Table 1) [13].

### Bleeding events and toxicity assessment

The grading of bleeding events was according to the World Health Organization (WHO) bleeding criteria [28]. The individual grades were defined in order: grade 0, no bleeding; grade 1, petechiae; grade 2, mild blood loss; grade 3, gross blood loss; grade 4, debilitating blood loss. The evaluation of CRS and ICANS was according to the American Society for Transplantation and Cellular Therapy guidelines [29]. Management of adverse events was based on guidelines and institutional experience [4, 30, 31].

### Statistical analysis

For uni-variate analysis, *χ*^2^ test or Fisher’s exact test was used for categorical data, and the Mann-Whitney *U* test was used for continuous variants. The before-and-after analysis of the same variable in the same patient was performed using a paired Wilcoxon test. Spearman correlation coefficients were used for correlations between continuous variables. The EASIX score was calculated as previously described [22]. The significance of cumulative event rates for ranked data was comparatively analyzed using the log rank test. Uni- and multi-variate analysis of categorical variables employed logistic regression. The cutoff values of risk factors were obtained by receiver operating characteristic (ROC) curves. *P* < 0.05 was considered statistically significant. Statistical analysis and figure drawing were generated by IBM SPSS statistics 26, and GraphPad Prism 8.

## Results

### Patient characteristics

A total of 56 patients were included in the analysis, with a median age of 44 (range, 13–74) years and 55.36% of male. Nineteen patients (33.93%) were diagnosed with B-ALL and 37 (66.07%) with NHL, including diffuse large B cell lymphoma, follicular lymphoma, mantle cell lymphoma, and B-cell lymphoblastic lymphoma. All patients received humanized anti-CD19 CAR T-cells. The median lines of previous treatment were 3 (range, 1–9), and 6 patients had received transplantation. The incidences of CRS and ICANS were 51.78% (*n*=29) and 7.14% (*n*=4), respectively. The incidences of severe (grade 3–4) CRS and ICANS were 10.91% (*n*=6) and 1.82% (*n*=1), respectively. All patient characteristics between bleeding and non-bleeding group were comparable (Table 1).

**Table 1:** Patient characteristics

Patient characteristics	Total (*n*=56)	Bleeding (*n*=17)	Non-bleeding (*n*=39)	*P* value
Sex, *n* (%)				0.81
Male	31 (55.36)	9 (52.94)	22 (56.41)	
Female	25 (44.64)	8 (47.06)	17 (43.59)	
Age, yr, median (range)	44 (13–74)	41 (17–74)	46 (13–72)	0.77
Diagnosis, *n* (%)				0.45
ALL	19 (33.93)	7 (41.18)	12 (30.77)	
NHL	37 (66.07)	10 (58.82)	27 (69.23)	
Prior transplant, *n* (%)	6 (10.71)	2 (11.76)	4 (10.26)	0.78
Prior lines of therapy, median (range)	3 (1–9)	3 (1–9)	3 (1–8)	0.62
CRS, *n* (%)				1.00
Grade 0–2	49 (89.09)	15 (88.24)	34 (89.47)	
Grade 3–4	6 (10.91)	2 (11.76)	4 (10.53)	
ICANS, *n* (%)				0.69
Grade 0–2	54 (98.18)	17 (100.00)	37 (97.37)	
Grade 3–4	1 (1.82)	0 (0)	1 (2.63)	

*ALL* acute lymphocytic leukemia, *NHL* non-Hodgkin lymphoma, *CRS* cytokine release syndrome, *ICANS* immune-effector cell-associated neurotoxicity syndrome

### Dynamic changes in coagulation indicators and severe coagulopathy

**Fig. 1:**
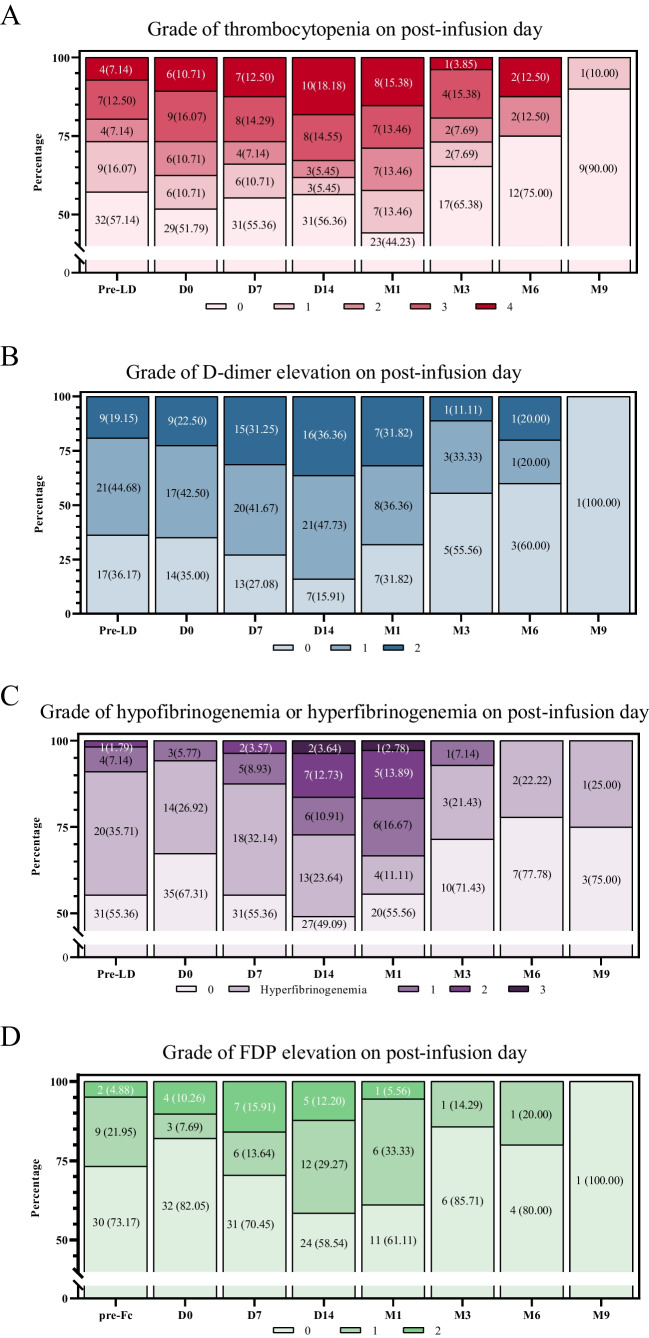
Dynamic changes in coagulation parameters after CAR T-cell therapy from pre-LD to M9. Percentage of patients with thrombocytopenia (**A**), D-dimer elevation (**B**), FIB abnormality (**C**), and FDP elevation (**D**) on post-infusion day. CAR chimeric antigen receptor, pre-LD pre-lymphodepletion, D day, M month, FIB fibrinogen, FDP fibrinogen degradation products

After CAR T-cell treatment, a significant proportion of patients developed severe coagulopathy. We found that percentages of ≥ grade 3 thrombocytopenia at pre-LD, D0, D7, D14, and M1 were 19.64%, 26.78%, 26.79%, 32.73%, and 28.84%, respectively (Fig. 1A). At pre-LD, D0, D7, D14, and M1, proportions of elevated D-dimer of >3 upper limits of normal (ULN) were 19.15%, 22.50%, 31.25%, 36.36%, and 31.82%, respectively (Fig. 1B). Hypofibrinogenemia of ≥ grade 3 occurred in 3.64% on D14 and 2.78% on M1 (Fig. 1C). Elevated FDP of >3 ULN occurred most frequently on D7 with the incidence rate of 15.91% (Fig. 1D). As other coagulation parameters, including APTT, TT, PT, INR, and ATIII fluctuated lightly, and details were shown in the supplemental Table 2, 3, 4, 5, 6. The majority of coagulation indicators changed most sharply around D7 and D14, with a significant difference from pre-LD, especially PLTs, D-dimer, APTT, PT, FDP, and ATIII (supplemental Figure 1 and 2).

Moreover, we analyzed the association between coagulation dysfunction and the development of CRS. In our study, patients with severe CRS had more dramatic changes in coagulation than those without or mild CRS (grade 0–2), especially on D14 (supplemental Figure 3 and 4).

### Cumulative incidence of bleeding events

**Fig. 2:**
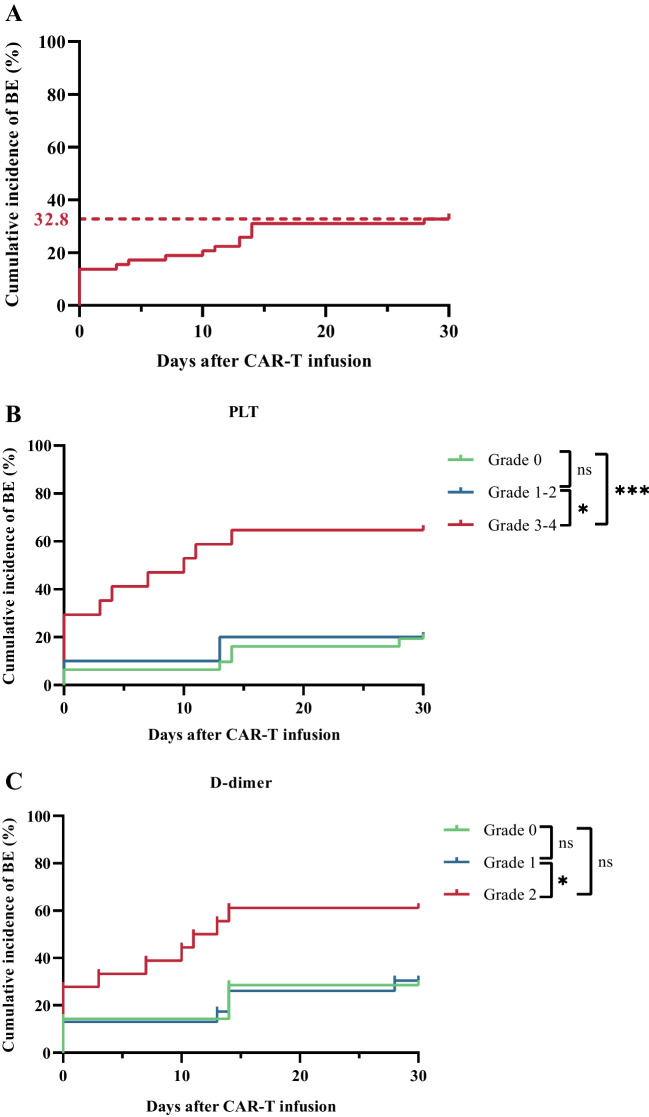
Cumulative incidence of bleeding event after CAR T-cell therapy. **A** The cumulative incidence of bleeding events within one month after CAR T-cell infusion was 32.8%. One patient had three bleeding episodes from different sites over 30 days. **B–C** Cumulative incidence of bleeding events by grade of thrombocytopenia on day 7 (**B**) and D-dimer elevation on day 14 (**C**). *P < 0.05. ***P < 0.001. ns no significance, CAR chimeric antigen receptor, BE bleeding events

The cumulative incidence of bleeding events within one month after infusion was 32.8%, with a median onset of 7 (range, 0–28) days (Fig. 2A). Based on the WHO bleeding criteria, all bleeding events were grade 1–3 (Table 2). Among them, the incidence of grade 2–3 bleeding events was 12.5%.

**Table 2:** Bleeding events within one month

Bleeding events	Total (*n*=56, %) *	WHO grade
Bloody pleural effusion	3 (5.36)	2
Occult blood in urine	6 (10.71)	1
Blood-tinged sputum	1 (1.79)	1
Oral mucosa bleed	2 (3.57)	2-3
Petechiae of skin	6 (10.71)	1
Gastrointestinal bleed	2 (3.57)	3
Epistaxis	1 (1.79)	2
Soft tissue bleed	1 (1.79)	2

*WHO* World Health Organization

*Three patients presented with 2 bleeding events at the same time, and one patient experienced three bleeding events at different times

Based on the dynamic changes of coagulation indicators after CAR T-cell infusion, we found that the correlation between the degree of abnormal PLTs on D7 or D-dimer on D14 and the cumulative incidence of bleeding events was statistically significant (Fig. 2B–C). However, we did not find a significant effect of CRS grades and bone-marrow tumor burden levels on the occurrence of bleeding events (supplemental Figure 5).

### Parameters associated with bleeding events

Supplemental Table 7 listed pre-LD, peak, and/or minimum values of monocyte, coagulation-, inflammation-, and tumor burden-related markers for patients with or without bleeding events. Patients with bleeding events had higher PT, IL-6, IL-10, and lower PLTs before lymphodepletion than those without bleeding events. Patients with bleeding events also had a higher peak of D-dimer, IL-2, IL-10, and lower monocyte nadir within one month.

### Correlation analysis of coagulation, inflammation, and tumor burden parameters

Peak D-dimer was weakly positively correlated with peak CRP (*r* = +0.36; 95% confidence interval [CI], 0.13–0.56; *P* = 0.007), while moderately positively related to peak IL-6 (*r* = +0.61; 95% CI, 0.40–0.75; *P* < 0.001), peak IL-10 (*r* = +0.57; 95% CI, 0.39–0.72; *P* < 0.001), peak ferritin (*r* = +0.67; 95% CI, 0.46–0.82; *P* < 0.001), and peak LDH (*r* = +0.75; 95% CI, 0.60–0.83; *P* < 0.001). Conversely, PLT nadir had a weak negative correlation with peak CRP (*r* = −0.27; 95% CI, −0.52– −0.01; *P* = 0.045), while showed moderately negative correlations with IL-6 (*r* = −0.41; 95% CI, −0.63– −0.13; *P* = 0.002), IL-10 (*r*= −0.56; 95% CI, −0.71– −0.36; *P* < 0.001), ferritin (*r* = −0.66; 95% CI, −0.82– −0.45; *P* < 0.001), LDH (*r* = −0.72; 95% CI, −0.82– −0.55; *P* < 0.001), and bone-marrow blast (*r*= −0.73; 95% CI, −0.90– −0.36; *P*=0.002), respectively. Except for the fact that there was no correlation between ATIII coagulation indicators and inflammatory-associated marker or LDH, all the other coagulation markers correlated to inflammation indicators or LDH.

**Fig. 3:**
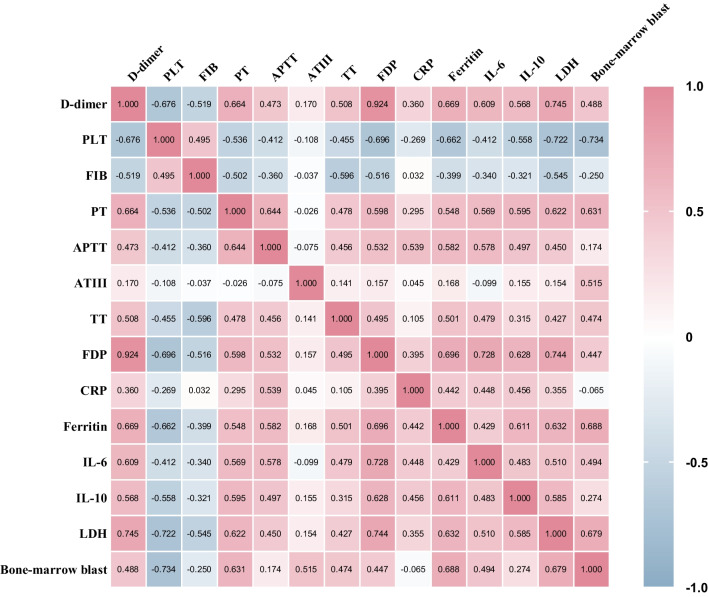
Correlations among coagulation-, inflammation-, and tumor burden-related markers. Spearman correlation coefficients were shown. *P* values for each correlation coefficient could be found in supplemental Table 8

Among these coagulation parameters, APTT was most strongly associated with the inflammatory factor CRP (*r* = +0.54; 95% CI, 0.29–0.73; *P* < 0.001). In addition, we also analyzed the correlation between bone-marrow blast and coagulation indicators in B-ALL patients, and found initial tumor burden was moderately positively correlated with peak PT (*r*= +0.63; 95% CI, 0.18–0.82; *P*=0.010) and peak ATIII (*r*= +0.5147; 95% CI, 0.01–0.81; *P*=0.044), while negatively correlated with PLT nadir. The full matrix of these parameters was illustrated in Fig. 3, most of which were statistically significant (*P* < 0.001) (supplemental Table 8).

### Predictors of bleeding events

**Fig. 4:**
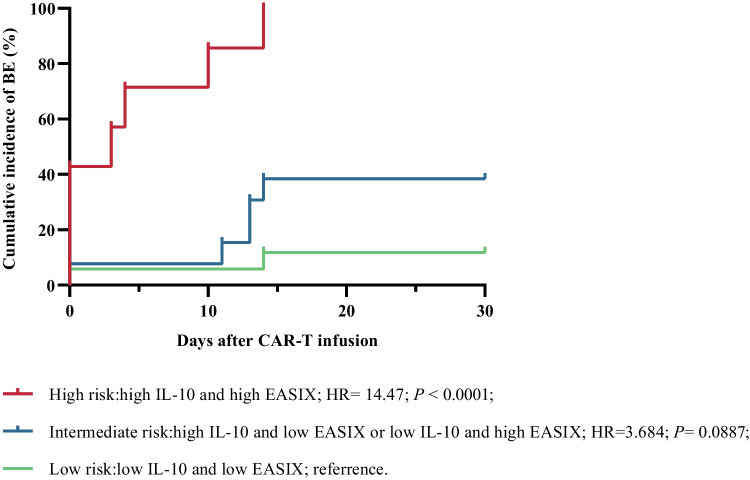
Cumulative incidence of bleeding events, according to IL-10 plus the EASIX score. High risk vs intermediate risk: HR, 5.50; 95% CI, 1.35–22.43; *P* = 0.0003. Only patients with available IL-10 levels were eligible for the analysis. IL interleukin, HR hazard ratio, CI confidence interval, BE bleeding events

Cutoff values of indicators were according to ROC curves shown in supplemental Figure 6. Patients with elevated pre-LD CRP (> 48.89 mg/L), IL-6 (> 7.49 pg/mL), IL-10 (> 7.98 pg/mL), the EASIX score (> 7.65), and PLT (< 104.50 ×10^9^/L) had a higher risk of bleeding events in uni-variate analysis (Table 3). In multi-variate analysis, elevated EASIX score and IL-10 before pretreatment could predict bleeding events. The odds ratio for bleeding events with IL-10 above 7.98 at baseline was 13.84 (95% CI, 2.03–94.36; *P* = 0.007), EASIX above 7.65 was 7.06 (95% CI, 1.03–48.23; *P* = 0.046) (Table 3). Risk stratification was based on IL-10 and the EASIX score. Patients in high-risk group characterized by IL-10 > 7.98 pg/mL and the EASIX score > 7.65 had a higher probability of bleeding (hazard ratio, 14.47; 95% CI, 2.78–75.29; *P* < 0.0001) (Fig. 4; supplemental Table 9). In addition, we also performed uni- and multivariate analysis on grade 2–3 bleeding events, and only the pre-LD EASIX score was statistically significant in multi-variate analysis (supplemental Table 10 and supplemental Figure 7).

**Table 3:** Univariate and multi-variate logistic regression analysis of bleeding events

Marker	Subgroup	No. case	No. non-case	Unadjusted OR (95% CI)	*P* value	Adjusted OR (95% CI)	*P* value
Pre-LD D-dimer (mg/L)	<1.13	8	25	1 (reference)	0.090		
	≥1.13	7	7	3.15 (0.84–11.65)			
Pre-LD CRP (mg/L)	≤48.89	11	34	1 (reference)	**0.021**		
	>48.89	6	3	6.18 (1.32–28.94)			
Pre-LD IL-6 (pg/ml)	≤7.49	2	14	1 (reference)	**0.008**		
	>7.49	12	8	10.50 (1.86–59.27)			
Pre-LD IL-10 (pg/ml)	≤7.98	5	19	1 (reference)	**0.006**	1 (reference)	**0.007**
	>7.98	8	3	10.13 (1.94–52.90)		13.84 (2.03–94.36)	
Pre-LD Ferritin (ng/ml)	≤654.60	4	15	1 (reference)	0.066		
	>654.60	10	10	3.75 (0.92–15.34)			
Pre-LD EASIX	≤7.65	6	27	1 (reference)	**0.014**	1 (reference)	**0.046**
	>7.65	9	8	5.06 (1.38–18.57)		7.06 (1.03–48.23)	
Pre-LD PLT (10^9^ /L)	≥104.50	5	25	1 (reference)	**0.021**		
	<104.50	12	14	4.29 (1.25–14.68)			
Pre-LD TNFα (pg/ml)	≤2.70	7	18	1 (reference)	0.085		
	>2.70	6	4	3.86 (0.83–17.94)			
CRS	≤0.50	6	20	1 (reference)	0.238		
	>0.50	11	18	2.04 (0.63–6.64)			

Bold indicated statistically significant results. *P* values in bold were less than 0.050

*OR* odds ratio, *LD* lymphodepletion, *CRP* C-reactive protein, *IL* interleukin, *EASIX* endothelial activation and stress index, *PLT* platelet, *TNFα* tumor necrosis factor α, *CRS* cytokine release syndrome

## Discussion

CARAC is a frequent adverse event during CAR T-cell treatment. Its occurrence is associated with myelosuppression, thrombocytopenia, and inflammation/tumor-mediated endothelial stress or injury [32]. Severe coagulopathy can lead to bleeding or thrombotic events, which can be fatal [11–13]. In the study, we analyzed dynamic changes in coagulation indicators, the cumulative incidence of bleeding events, correlations among coagulation, inflammation, and tumor burden in a small cohort of 56 patients. Finally, we screened two statistically significant variates, IL-10 and the EASIX score, and constructed a risk-stratification model for predicting bleeding events after CAR T-cell therapy.

We comprehensively analyzed the dynamic changes of routine coagulation indicators from pre-LD to M9 after CAR T-cell treatment. Long-term follow-up results showed that abnormalities in coagulation function mainly occurred within one month and were alleviated about 3 months after treatment. Thrombocytopenia occurred frequently and severely on D14 and gradually recovered thereafter. The abnormal proportions of APTT, FDP, ATIII, TT, and INR were relatively small, but there were obvious abnormal peaks on D7 and D14, which was consistent with previous studies [8, 10]. In addition, the severity of PLTs and D-dimer fluctuation on D7 and D14 is correlated to the occurrence of bleeding events. Many previous studies had also used D-dimer as a marker for predicting bleeding events [18, 33]. These findings prompted us to closely monitor the coagulation function of patients within one month after infusion, especially within the first two weeks.

We analyzed pre-LD, peak, and/or minimum clinical indicators within one month post infusion between bleeding and non-bleeding groups. Patients in bleeding group had lower pre-LD PLTs than those in non-bleeding group (*P* = 0.022), consistent with the previous study [13]. Bone-marrow infiltration or impaired hematopoietic function due to multi-lines of previous therapies might be responsible for pre-LD thrombocytopenia, as well as the abnormalities of PLT function [34, 35]. PLTs played an important role in vascular endothelial generation and endothelial integrity maintenance [36]. Thrombocytopenia would hinder the differentiation of endothelial progenitor cells to mature endothelial cells and the migration of mature endothelial cells [37, 38]. Decreased number and impaired function of PLTs led to the reduced secretion of proangiogenic cytokines and growth factors, further resulted in molecular degradation between adjacent endothelial cells and disruption of endotheliocyte homeostasis and integrity [39]. Petechiae in some patients formed when red blood cells leaked from the destroyed endothelial gap to the interstitial space. Moreover, endothelial homeostasis imbalances lead to disturbances of the endogenous and exogenous coagulation systems. Furthermore, von Willebrand factor (vWF), tissue factor, and e-selectin are markers of endothelial activation, which can be included in subsequent studies [40, 41]. We also observed lower monocyte nadir in patients with bleeding, as mentioned by Johnsrud et al [13]. Strati et al. [42] illustrated that patient with grade 3–4 ICANS were characterized by lower monocytes perhaps due to monocytes migration from the blood to central nervous tissue and mediating inflammatory effects. Similarly, monocytes might exude from the vascular endothelial space along with red blood cells when bleeding events occurred.

Studies had demonstrated that clotting, inflammation, and tumors interact with each other [13, 17–19]. The conclusions of our study also supported the above point of view. Endothelial cell activation mediated by tumor cells could induce the transformation of cell surfaces into adherent and pro-coagulable surfaces, which in turn affected the homeostasis of the coagulation system [43]. Some inflammatory molecules could activate the endothelium and promote coagulation, and some factors in the coagulation system were also effective inflammatory mediators. Moreover, CRS and ICANS during CAR T-cell treatment were systemic inflammatory responses, and their occurrence had been shown to affect coagulation function [10]. However, CRS and ICANS were not inevitable factors for bleeding. We did not find differences in the incidence of CRS and ICANS between bleeders and non-bleeders. The study by Johnsrud et al. found that the incidence of ICANS was significantly higher in the bleeding group than in the non-bleeding group in uni-variate analysis, while CRS was not different between the two groups. However, there was no difference in ICANS between the two groups after multi-variate analysis, indicating that CRS and ICANS were not independent risk factors for hemorrhage [13]. Therefore, it is necessary to pay attention to the inflammation, tumor burden, and coagulation function, thus to adopt treatment strategies in managing coagulation disorders.

Several variants of EASIX had been created to predict CRS and ICANS in CAR T-cell therapy, including simplified EASIX (s-EASIX, LDH/ PLT), modified EASIX (m-EASIX, LDH × CRP /PLT), or combined with ferritin and/or CRS for risk stratification [23–25]. Further studies needed to be explored to create more preferable EASIX score for the prediction of coagulation and hemorrhage. When analyzing high-risk factors for bleeding events, the uni-variate analysis included major coagulation-, inflammatory-, and tumor burden-related parameters. However, only the EASIX score and IL-10 were significant in the multi-variate analysis model. The EASIX, defined as LDH × creatinine / PLT, indicated the endothelial activation and stress [22]. A study had shown that hypercoagulation status characterized by significantly increased factor VIII, factor XI, vWF antigen, vWF ristocetin, ATIII, and FIB was associated with endothelial cell activation, irrespective of disease state [44]. In addition, disruption of endothelial integrity and loss of vasoconstriction can lead to hemorrhage. Patients with EASIX scores higher than 7.65 and IL-10 greater than 7.98 pg/ml before lymphodepletion were at higher risk of hemorrhage, and their coagulation function during treatment should be closely monitored and preventive measures should be taken in a timely manner to prevent serious bleeding events. IL-10 was an anti-inflammatory cytokine that inhibits inflammation and promotes repair. Elevated IL-10 might represent imbalances of the immune system and inflammatory system, acting as an “alarm signal.” Enhanced IL-10 promoted apoptosis by inhibiting intravenous endothelial proliferation via the STAT3 pathway [45]. However, IL-10 had also been reported to promote endothelial progenitor cell infiltration and wound healing via the STAT3 pathway [46]. Perhaps the effect of IL-10 is affected by concentration. In patients with severe COVID-19, IL-10 was negatively correlated with vWF, which indicated endothelium dysfunction [41]. The current studies have mostly focused on IL-6 [47], and the role of IL-10 in endothelial homeostasis and coagulation disorders needed further investigation.

Compared with other predictive models, the inclusion of the EASIX score and IL-10, which are routine clinical monitoring indicators, has improved clinical practicality. Moreover, we included the EASIX for the first time in the analysis of hemorrhage after CAR T-cell therapy. However, this study was based on a single-center small cohort and needed to be validated in a larger population.

## Conclusion

In summary, we described the changes in coagulation function within one month after CAR T-cell infusion. By multi-variate logistic regression, we finally included the EASIX score and IL-10 as statistically significant predictors to set a risk-stratification model for hemorrhage after CAR T-cell therapy. Risk factors for bleeding events should be further prospectively explored and strategies for their prevention and management are needed to be developed.

### Supplementary information

ESM 1 (PDF 523 kb)

## Data Availability

All data in the study were included in the article and supplemental materials.

## References

[1] Locke FL, Ghobadi A, Jacobson CA, Miklos DB, Lekakis LJ, Oluwole OO, Lin Y, Braunschweig I, Hill BT, Timmerman JM, Deol A, Reagan PM, Stiff P, Flinn IW, Farooq U, Goy A, McSsweeney PA, Munoz J, Siddiqi T, Chavez JC, Herrera AF, Bartlett NL, Wiezorek JS, Navale L, Xue A, Jiang Y, Bot A, Rossi JM, Kim JJ, Go WY, Neelapu SS (2019). Long-term safety and activity of axicabtagene ciloleucel in refractory large B-cell lymphoma (ZUMA-1): a single-arm, multicentre, phase 1–2 trial. Lancet Oncol.

[2] Park JH, Rivière I, Gonen M, Wang X, Sénéchal B, Curran KJ, Sauter C, Wang Y, Santomasso B, Mead E, Roshal M, Maslak P, Davila M, Brentjens RJ, Sadelain M (2018). Long-term follow-up of CD19 CAR therapy in acute lymphoblastic leukemia. N Engl J Med.

[3] Maude SL, Laetsch TW, Buechner J, Rives S, Boyer M, Bittencourt H, Bader P, Verneris MR, Stefanski HE, Myers GD, Qayed M, De Moerloose B, Hiramatsu H, Schlis K, Davis KL, Martin PL, Nemecek ER, Yanik GA, Peters C, Baruchel A, Boissel N, Mechinaud F, Balduzzi A, Krueger J, June CH, Levine BL, Wood P, Taran T, Leung M, Mueller KT, Zhang Y, Sen K, Lebwohl D, Pulsipher MA, Grupp SA (2018). Tisagenlecleucel in children and young adults with B-cell lymphoblastic leukemia. New Engl J Med.

[4] Mei H, Chen F, Han Y, Hou M, Huang H, Huang X, Li Y, Liang A, Liu Q, Niu T, Peng J, Qian W, Song Y, Wang J, Wang Y, Wu D, Xu K, Yang L, Yang R, Zhang L, Zhang L, Zhang X, Zhang X, Zhao W, Han W, Hu Y (2022). Chinese expert consensus on the management of chimeric antigen receptor T cell therapy-associated coagulopathy. Chin Med J.

[5] Yamasaki-Morita M, Arai Y, Ishihara T, Onishi T, Shimo H, Nakanishi K, Nishiyama Y, Jo T, Hiramatsu H, Mitsuyoshi T, Mizumoto C, Kanda J, Nishikori M, Kitawaki T, Nogami K, Takaori-Kondo A, Nagao M, Adachi S (2022). Relative hypercoagulation induced by suppressed fibrinolysis after tisagenlecleucel infusion in malignant lymphoma. Blood Adv.

[6] Ishihara T, Arai Y, Morita M, Onishi T, Shimo H, Kitawaki T, Takaori-Kondo A, Adachi S, Nogami K (2021). Suppressed fibrinolytic activity demonstrated by simultaneous thrombin and plasmin generation assay during cytokine release syndrome after CD19 chimeric antigen receptor-modified T-cell therapy. Blood.

[7] Belbachir S, Tudesq JJ, Lamure S, Rocanieres P, Properzi E, Ceballos P, Fegueux N, Gehlkopf E, Paul F, Tchernonog E, Theron A, Teyssier A-C, Diaz I, Patricia A-M, Herbaux C, Cartron G (2021). Coagulopathy following chimeric antigen receptor T cell therapy in R/R adult B cell malignancies: a single center experience. Blood.

[8] Wang Y, Qi K, Cheng H, Cao J, Shi M, Qiao J, Yan Z, Jing G, Pan B, Sang W, Li D, Wang X, Fu C, Zhu F, Zheng J, Li Z, Xu K (2020). Coagulation disorders after chimeric antigen receptor T cell therapy: analysis of 100 patients with relapsed and refractory hematologic malignancies. Biol Blood Marrow Transplant.

[9] Mei H, Jiang H, Wu Y, Guo T, Xia L, Jin R, Hu Y (2018). Neurological toxicities and coagulation disorders in the cytokine release syndrome during CAR-T therapy. Br J Haematol.

[10] Shao M, Yu Q, Teng X, Guo X, Wei G, Xu H, Cui J, Chang AH, Hu Y, Huang H (2021). CRS-related coagulopathy in BCMA targeted CAR-T therapy: a retrospective analysis in a phase I/II clinical trial. Bone Marrow Transplant.

[11] Yan Z, Cao J, Cheng H, Qiao J, Zhang H, Wang Y, Shi M, Lan J, Fei X, Jin L, Jing G, Sang W, Zhu F, Chen W, Wu Q, Yao Y, Wang G, Zhao J, Zheng J, Li Z, Xu K (2019). A combination of humanised anti-CD19 and anti-BCMA CAR T cells in patients with relapsed or refractory multiple myeloma: a single-arm, phase 2 trial. Lancet Haematol.

[12] Pan J, Tan Y, Wang G, Deng B, Ling Z, Song W, Seery S, Zhang Y, Peng S, Xu J, Duan J, Wang Z, Yu X, Zheng Q, Xu X, Yuan Y, Yan F, Tian Z, Tang K, Zhang J, Chang AH, Feng X (2021). Donor-derived CD7 chimeric antigen receptor T cells for T-cell acute lymphoblastic leukemia: first-in-human, phase I trial. J Clin Oncol.

[13] Johnsrud A, Craig J, Baird J, Spiegel J, Muffly L, Zehnder J, Tamaresis J, Negrin R, Johnston L, Arai S, Shizuru J, Lowsky R, Meyer E, Weng WK, Shiraz P, Rezvani A, Latchford T, Mackall C, Miklos D, Frank M, Sidana S (2021). Incidence and risk factors associated with bleeding and thrombosis following chimeric antigen receptor T-cell therapy. Blood Adv.

[14] Hashmi H, Mirza AS, Darwin A, Logothetis C, Garcia F, Kommalapati A, Mhaskar RS, Bachmeier C, Chavez JC, Shah B, Pinilla-Ibarz J, Khimani F, Lazaryan A, Liu H, Davila ML, Locke FL, Nishihori T, Jain MD (2020). Venous thromboembolism associated with CD19-directed CAR T-cell therapy in large B-cell lymphoma. Blood Adv.

[15] Jiang H, Liu L, Guo T, Wu Y, Ai L, Deng J, Dong J, Mei H, Hu Y (2019). Improving the safety of CAR-T cell therapy by controlling CRS-related coagulopathy. Ann Hematol.

[16] Qi J, Lv X, Chen J, Wang H, Chu T, Tang Y, Pan T, Zhou M, Cai C, Ren Y, Liu Y, Fan Y, Shen W, Ma X, Qiu H, Tang X, Fu C, Wu D, Han Y (2022). TNF-alpha increases the risk of bleeding in patients after CAR T-cell therapy: a bleeding model based on a real-world study of Chinese CAR T Working Party. Hematol Oncol.

[17] Locke FL, Rossi JM, Neelapu SS, Jacobson CA, Miklos DB, Ghobadi A, Oluwole OO, Reagan PM, Lekakis LJ, Lin Y, Sherman M, Better M, Go WY, Wiezorek JS, Xue A, Bot A (2020). Tumor burden, inflammation, and product attributes determine outcomes of axicabtagene ciloleucel in large B-cell lymphoma. Blood Adv.

[18] Al-Samkari H, Karp Leaf RS, Dzik WH, Carlson JCT, Fogerty AE, Waheed A, Goodarzi K, Bendapudi PK, Bornikova L, Gupta S, Leaf DE, Kuter DJ, Rosovsky RP (2020). COVID-19 and coagulation: bleeding and thrombotic manifestations of SARS-CoV-2 infection. Blood.

[19] Zhou S, Chen W, Lin M, Chen G, Chen C, Huo C, Du X (2021). Correlation of 18F-FDG PET/CT SUVmax with clinical features D-dimer and LDH in patients with primary intestinal lymphoma. J Int Med Res.

[20] van Hinsbergh VW (2012). Endothelium—role in regulation of coagulation and inflammation. Semin Immunopathol.

[21] Ruutu T, Barosi G, Benjamin RJ, Clark RE, George JN, Gratwohl A, Holler E, Iacobelli M, Kentouche K, Lämmle B, Moake JL, Richardson P, Socié G, Zeigler Z, Niederwieser D, Barbui T (2007). Diagnostic criteria for hematopoietic stem cell transplant-associated microangiopathy: results of a consensus process by an International Working Group. Haematologica.

[22] Luft T, Benner A, Jodele S, Dandoy CE, Storb R, Gooley T, Sandmaier BM, Becker N, Radujkovic A, Dreger P, Penack O (2017). EASIX in patients with acute graft-versus-host disease: a retrospective cohort analysis. Lancet Haematol.

[23] Pennisi M, Sanchez-Escamilla M, Flynn JR, Shouval R, Alarcon Tomas A, Silverberg ML, Batlevi C, Brentjens RJ, Dahi PB, Devlin SM, Diamonte C, Giralt S, Halton EF, Jain T, Maloy M, Mead E, Palomba ML, Ruiz J, Santomasso B, Sauter CS, Scordo M, Shah GL, Park JH, Segundo LYS, Perales MA (2021). Modified EASIX predicts severe cytokine release syndrome and neurotoxicity after chimeric antigen receptor T cells. Blood Adv.

[24] Korell F, Penack O, Mattie M, Schreck N, Benner A, Krzykalla J, Wang Z, Schmitt M, Bullinger L, Muller-Tidow C, Dreger P, Luft T (2022). EASIX and severe endothelial complications after CD19-directed CAR-T cell therapy—a cohort study. Front Immunol.

[25] Greenbaum U, Strati P, Saliba RM, Torres J, Rondon G, Nieto Y, Hosing C, Srour SA, Westin J, Fayad LE, Lee HJ, Iyer SP, Nair R, Nastoupil LJ, Parmar S, Rodriguez MA, Samaniego F, Steiner RE, Wang M, Pinnix CC, Flowers CR, Tummala S, Ramdial JL, Yalniz FF, Hawkins M, Rezvani K, Champlin RE, Shpall EJ, Neelapu SS, Kebriaei P, Ahmed S (2021). CRP and ferritin in addition to the EASIX score predict CAR-T-related toxicity. Blood Adv.

[26] Zhang Y, Zhou F, Wu Z, Li Y, Li C, Du M, Luo W, Kou H, Lu C, Mei H (2022). Timing of tocilizumab administration under the guidance of IL-6 in CAR-T therapy for R/R acute lymphoblastic leukemia. Front Immunol.

[27] Luo W, Li C, Wu J, Tang L, Wang X, Zhang Y, Wu Z, Huang Z, Xu J, Kang Y, Xiong W, Deng J, Hu Y, Mei H (2023). Bruton tyrosine kinase inhibitors preserve anti-CD19 chimeric antigen receptor T-cell functionality and reprogram tumor micro-environment in B-cell lymphoma. Cytotherapy.

[28] Miller AB, Hoogstraten B, Staquet M, Winkler A (1981). Reporting results of cancer treatment. Cancer.

[29] Lee DW, Santomasso BD, Locke FL, Ghobadi A, Turtle CJ, Brudno JN, Maus MV, Park JH, Mead E, Pavletic S, Go WY, Eldjerou L, Gardner RA, Frey N, Curran KJ, Peggs K, Pasquini M, DiPersio JF, van den Brink MRM, Komanduri KV, Grupp SA, Neelapu SS (2019). ASTCT consensus grading for cytokine release syndrome and neurologic toxicity associated with immune effector cells. Biol Blood Marrow Transplant.

[30] Buechner J, Grupp SA, Hiramatsu H, Teachey DT, Rives S, Laetsch TW, Yanik GA, Wood P, Awasthi R, Yi L, Chassot-Agostinho A, Eldjerou LK, De Moerloose B (2021). Practical guidelines for monitoring and management of coagulopathy following tisagenlecleucel CAR T-cell therapy. Blood Adv.

[31] Santomasso BD, Nastoupil LJ, Adkins S, Lacchetti C, Schneider BJ, Anadkat M, Atkins MB, Brassil KJ, Caterino JM, Chau I, Davies MJ, Ernstoff MS, Fecher L, Funchain P, Jaiyesimi I, Mammen JS, Naidoo J, Naing A, Phillips T, Porter LD, Reichner CA, Seigel C, Song JM, Spira A, Suarez-Almazor M, Swami U, Thompson JA, Vikas P, Wang Y, Weber JS, Bollin K, Ghosh M (2021). Management of immune-related adverse events in patients treated with chimeric antigen receptor T-cell therapy: ASCO guideline. J Clin Oncol.

[32] Juluri KR, Wu QV, Voutsinas J, Hou J, Hirayama AV, Mullane E, Miles N, Maloney DG, Turtle CJ, Bar M, Gauthier J (2022). Severe cytokine release syndrome is associated with hematologic toxicity following CD19 CAR T-cell therapy. Blood Adv.

[33] Otani T, Sawano H, Natsukawa T, Matsuoka R, Nakashima T, Takahagi M, Hayashi Y (2018). D-dimer predicts bleeding complication in out-of-hospital cardiac arrest resuscitated with ECMO. Am J Emerg Med.

[34] Nissani A, Lev-Ari S, Meirson T, Jacoby E, Asher N, Ben-Betzalel G, Itzhaki O, Shapira-Frommer R, Schachter J, Markel G, Besser MJ (2021) Comparison of non-myeloablative lymphodepleting preconditioning regimens in patients undergoing adoptive T cell therapy. J Immunother Cancer 9(5):e001743 10.1136/jitc-2020-001743 PMC8127974 33990415

[35] Ferreyro BL, Scales DC, Wunsch H, Cheung MC, Gupta V, Saskin R, Thyagu S, Munshi L (2021). Critical illness in patients with hematologic malignancy: a population-based cohort study. Intensive Care Med.

[36] Nachman RL, Rafii S (2008). Platelets, petechiae, and preservation of the vascular wall. N Engl J Med.

[37] Sun Y, Liu XL, Zhang D, Liu F, Cheng YJ, Ma Y, Zhou YJ, Zhao YX (2019). Platelet-derived exosomes affect the proliferation and migration of human umbilical vein endothelial cells via miR-126. Curr Vasc Pharmacol.

[38] Cadamuro M, Brivio S, Mertens J, Vismara M, Moncsek A, Milani C, Fingas C, Cristina Malerba M, Nardo G, Dall’Olmo L, Milani E, Mariotti V, Stecca T, Massani M, Spirli C, Fiorotto R, Indraccolo S, Strazzabosco M, Fabris L (2019). Platelet-derived growth factor-D enables liver myofibroblasts to promote tumor lymphangiogenesis in cholangiocarcinoma. J Hepatol.

[39] Zhang Y, Cedervall J, Hamidi A, Herre M, Viitaniemi K, D’Amico G, Miao Z, Unnithan RVM, Vaccaro A, van Hooren L, Georganaki M, Thulin Å, Qiao Q, Andrae J, Siegbahn A, Heldin CH, Alitalo K, Betsholtz C, Dimberg A, Olsson AK (2020). Platelet-specific PDGFB ablation impairs tumor vessel integrity and promotes metastasis. Cancer Res.

[40] Langstrom S, Koskenvuo M, Huttunen P, Lassila R, Taskinen M, Ranta S, Heikinheimo M, Makipernaa A (2018). Haematopoietic stem cell transplantation in children shifts the coagulation system towards a pro-coagulant state. Thromb Haemost.

[41] Hokama LT, Veiga ADM, Menezes MCS, Sardinha Pinto AA, de Lima TM, Ariga SKK, Barbeiro HV, Barbeiro DF, de Lucena Moreira C, Stanzani G, Brandao RA, Marchini JF, Alencar JC, Marino LO, Gomez LM, U.S.P.C.-G. Emergency, H.P. Souza (2022). Endothelial injury in COVID-19 and septic patients. Microvasc Res.

[42] Strati P, Nastoupil LJ, Westin J, Fayad LE, Ahmed S, Fowler NH, Hagemeister FB, Lee HJ, Iyer SP, Nair R, Parmar S, Rodriguez MA, Samaniego F, Steiner RE, Wang M, Pinnix CC, Adkins S, Claussen CM, Martinez CS, Hawkins MC, Johnson NA, Singh P, Mistry HE, Horowitz S, George S, Feng L, Kebriaei P, Shpall EJ, Neelapu SS, Tummala S, Chi TL (2020). Clinical and radiologic correlates of neurotoxicity after axicabtagene ciloleucel in large B-cell lymphoma. Blood Adv.

[43] Wagner DD, Frenette PS (2008). The vessel wall and its interactions. Blood.

[44] Probst K, Fijnheer R, Rothova A (2004). Endothelial cell activation and hypercoagulability in ocular Behcet's disease. Am J Ophthalmol.

[45] Xie Z, Lin B, Jia X, Su T, Wei Y, Tang J, Yang C, Cui C, Liu J (2021). Enhanced IL-10 inhibits proliferation and promotes apoptosis of HUVECs through STAT3 signaling pathway in sepsis. Histol Histopathol.

[46] Short WD, Steen E, Kaul A, Wang X, Olutoye OO, Vangapandu HV, Templeman N, Blum AJ, Moles CM, Narmoneva DA, Crombleholme TM, Butte MJ, Bollyky PL, Keswani SG, Balaji S (2022). IL-10 promotes endothelial progenitor cell infiltration and wound healing via STAT3. FASEB J.

[47] Kang S, Tanaka T, Inoue H, Ono C, Hashimoto S, Kioi Y, Matsumoto H, Matsuura H, Matsubara T, Shimizu K, Ogura H, Matsuura Y, Kishimoto T (2020). IL-6 trans-signaling induces plasminogen activator inhibitor-1 from vascular endothelial cells in cytokine release syndrome. Proc Natl Acad Sci U S A.

