# Supplementary material for: IL-10 plus the EASIX score predict bleeding events after anti-CD19 CAR T-cell therapy
Source: Ann Hematol. 2023 Oct 9;102(12):3575–85. doi: 10.1007/s00277-023-05477-y (PMC10640490; doi:10.1007/s00277-023-05477-y)
Supplement: Supplementary file 1 — (PDF 523 kb) [file 277_2023_5477_MOESM1_ESM.pdf]

Supplementary Material for

IL-10 plus the EASIX score predict bleeding events after anti-CD19 CAR T-cell therapy

Xindi Wang,[1,2]* Chenggong Li,[1,2]* Wenjing Luo,[1,2] Yinqiang Zhang,[1,2] Zhongpei Huang,[1,2] Jia Xu,[1,2] Heng Mei,[1,2#] and Yu Hu[1,2#]

1. Institute of Hematology, Union Hospital, Tongji Medical College, Huazhong University of Science and Technology, Wuhan, 430022, China.
2. Hubei Clinical Medical Center of Cell Therapy for Neoplastic Disease, Wuhan, 430022, China.

*XW and CL contributed equally as co-first authors.

#HM and YH contributed equally as co-senior authors.

The PDF file includes:

Supplementary Table 1 to 10;

Supplementary Figure 1 to 7

Supplemental Table 1. Grading of the coagulation parameters

Parameter	Grade 1	Grade 2	Grade 3	Grade 4
**PLT**	< LLN, ≥75	<75, ≥50	<50, ≥25	<25
**FIB**	0.75-1 LLN	0.5- 0.75 LLN	0.25- 0.5 LLN	<0.25 LLN
**INR**	≤1.5 ULN	>1.5-2.5 ULN	>2.5 ULN	NA
**APTT**	≤1.5 ULN	>1.5-2.5 ULN	>2.5 ULN	NA
**D-dimer**	1-3 ULN	>3 ULN	NA	NA
**PT**	Prolong ≤3s	Prolong > 3s	NA	NA
**ATIII**	0.75-1 LLN	0.5- 0.75 LLN	0.25- 0.5 LLN	<0.25 LLN
**FDP**	1-3 ULN	>3 ULN	NA	NA
**TT**	Abnormal	NA	NA	NA

PLT indicates platelet; FIB, fibrinogen; INR, international normalized ratio; APTT, activated partial thromboplastin time; PT, prothrombin time; ATIII, antithrombin III; FDP, fibrinogen degradation products; TT, thrombin time; LLN, lower limit of normal; ULN, upper limit of normal; NA, not application.

Supplemental Table 2. Grade of APTT prolongation on post-infusion day

Grade	Pre-LD	D0	D7	D14	M1	M3	M6	M9
**Grade 0 (n, %)**	49 (87.5)	41 (78.85)	42 (75)	46 (83.64)	34 (94.44)	12 (85.71)	9 (100)	4 (100)
**Grade 1 (n, %)**	4 (7.14)	8 (15.38)	6 (10.71)	5 (9.09)	1 (2.78)	1 (7.14)	0 (0)	0 (0)
**Grade 2 (n, %)**	3 (5.36)	3 (5.77)	8 (14.29)	4 (7.27)	1 (2.78)	1 (7.14)	0 (0)	0 (0)
**Total (n)**	56	52	56	55	36	14	9	4

APTT indicates activated partial thromboplastin time; Pre-LD, pre-lymphodepletion; D, day; M, month.

Supplemental Table 3. Grade of TT prolongation on post-infusion day

Grade	Pre-LD	D0	D7	D14	M1	M3	M6	M9
**Normal (n, %)**	56 (100)	51 (98.08)	54 (96.43)	47 (85.45)	34 (94.44)	14 (100)	9 (100)	4 (100)
**Abnormal (n, %)**	0 (0)	1 (1.92)	2 (3.58)	8 (14.55)	2 (5.56)	0 (0)	0 (0)	0 (0)
**Total (n)**	56	52	56	55	36	14	9	4

TT indicates thrombin time; Pre-LD, pre-lymphodepletion; D, day; M, month.

Supplemental Table 4. Grade of ATIII elevation on post-infusion day

Grade	Pre-LD	D0	D7	D14	M1	M3	M6	M9
**Grade 0 (n, %)**	30 (73.17)	35 (87.5)	36 (80)	32 (80)	15 (83.33)	7 (100)	4 (80)	1 (100)
**Grade 1 (n, %)**	9 (21.95)	4 (10)	7 (15.56)	7 (17.5)	2 (11.11)	0 (0)	1 (20)	0 (0)
**Grade 2 (n, %)**	2 (4.88)	0 (0)	2 (4.44)	1 (2.5)	1 (5.56)	0 (0)	0 (0)	0 (0)
**Grade 3 (n, %)**	0 (0)	1 (2.5)	0 (0)	0 (0)	0 (0)	0 (0)	0 (0)	0 (0)
**Total (n)**	41	40	45	40	18	7	5	1

ATIII indicates antithrombin III; Pre-LD, pre-lymphodepletion; D, day; M, month.

Supplemental Table 5. Grade of PT prolongation on post-infusion day

Grade	Pre-LD	D0	D7	D14	M1	M3	M6	M9
**Grade 0 (n, %)**	53 (94.64)	51 (98.08)	51 (91.07)	52 (94.55)	34 (94.44)	13 (100)	9 (100)	4 (100)
**Grade 1 (n, %)**	3 (5.36)	1 (1.92)	4 (7.14)	3 (5.45)	2 (5.56)	0 (0)	0 (0)	0 (0)
**Grade 2 (n, %)**	0 (0)	0 (0)	1 (1.79)	0 (0)	0 (0)	0 (0)	0 (0)	0 (0)
**Total (n)**	56	52	56	55	36	13	9	4

PT indicates prothrombin time; Pre-LD, pre-lymphodepletion; D, day; M, month.

Supplemental Table 6. Grade of INR elevation on post-infusion day

Grade	Pre-LD	D0	D7	D14	M1	M3	M6	M9
**Grade 0 (n, %)**	53 (94.64)	51 (98.08)	50 (89.29)	51(92.73)	33(91.67)	14(100)	9(100)	4(100)
**Grade 1 (n, %)**	3(5.36)	1(1.92)	5(8.93)	4(7.27)	3(8.33)	0(0)	0(0)	0(0)
**Grade 2 (n, %)**	0(0)	0(0)	1(1.79)	0(0)	0(0)	0(0)	0(0)	0(0)
**Total (n)**	56	52	56	55	36	14	9	4

INR indicates international normalized ratio; Pre-LD, pre-lymphodepletion; D, day; M, month.

Supplemental Table 7. Coagulation, inflammatory, and tumor burden markers by bleeding events

Laboratory findings	Total	Non-bleeding	Bleeding	P value (non-bleeding vs bleeding)
**PLT (×10^9/L)**				
Pre-LD	115.50 (67.75–197.75)	131.00 (82.00–210.00)	82.00 (29.5–142.5)	**0.022**
Minimum	49.0 (16.2–105.5)	50.50 (17.75–114.75)	22.50 (10.75–83.25)	0.056
**D-dimer (mg/L)**				
Pre-LD	0.66 (0.37–1.22)	0.64 (0.36–1.06)	0.67 (0.38–2.45)	0.373
Peak	1.59 (0.81–4.62)	0.96 (0.71–4.96)	2.42 (1.16–11.97)	**0.040**
**APTT (s)**				
Pre-LD	37.00 (34.10–40.90)	37.05 (34.38–40.60)	37.00 (33.70–42.65)	0.827
Peak	43.70 (37.26–50.50)	46.40 (37.35–51.28)	43.45 (40.60–60.33)	0.993
**FIB (g/l)**				
Pre-LD	3.22 (2.52–4.24)	3.15 (2.30–4.16)	3.43 (2.77–4.85)	0.348
Minimum	2.32 (1.47–3.30)	2.29 (1.65–3.00)	2.31 (1.11–3.43)	0.599
Peak	4.00 (3.21–4.90)	4.03 (3.12–4.72)	4.13 (3.46–5.52)	0.859
**FDP (ug/ml)**				
Pre-LD	4.00 (4.00–5.10)	4.00 (4.00–4.55)	4.00 (2.50–7.80)	0.738
Peak	5.50 (4.08–17.25)	4.65 (4.00 – 15.75)	7.25 (4.75–50.35)	0.103
**TT (s)**				
Pre-LD	17.00 (15.80–18.00)	17.00 (16.05–18.00)	16.50 (15.70–18.15)	0.964
Peak	18.60 (17.30–21.75)	18.58 (16.73–21.73)	18.45 (17.48–26.65)	0.556
**ATIII (%)**				
Pre-LD	89.00 (77.50–96.00)	92.00 (79.00–96.00)	81.00(65.5–94.5)	0.120
Peak	104.00 (94.88–110.50)	103.50 (94.75–110.00)	103.50(94.00–112.50)	0.712
**PT (s)**				
Pre-LD	13.30 (13.00–13.80)	13.20 (12.80–13.73)	13.70 (13.25–14.55)	**0.014**
Peak	14.07 (13.38–15.55)	13.95 (13.18–15.35)	14.50 (13.58–17.23)	0.076
**Monocyte (×10^9/L)**				

Pre-LD	0.30 (0.16–0.48)	0.27 (0.13–0.44)	0.30 (0.21–0.68)	0.123
Minimum	0.02 (0.0–0.06)	0.02 (0.00–0.06)	0.00 (0.00–0.02)	**0.017**
Peak	0.41 (0.24–0.63)	0.44 (0.24–0.60)	0.29 (0.17–0.50)	0.212
**Ferritin (ng/ml)**				
Pre-LD	654.55 (234.88–1310.88)	552.35 (174.27–1168.57)	865.55 (445.98–2293.48)	0.204
Peak	1420.80 (431.23–5558.35)	1470.90 (372.50–10528.88)	1721.80 (578.08–5798.55)	0.795
**LDH (U/L)**				
Pre-LD	242.00 (194.25–494.50)	224.00 (181.00–327.00)	304.00 (209.00–719.00)	0.105
Peak	312.00 (225.50–1050.50)	279.50 (221.25–853.00)	607.50 (238.50–2259.25)	0.073
**Tumor burden (%)**				
Pre-LD	32.25 (4.00–96.50)	21.50 (5.54–91.00)	66.50 (4.00–96.50)	0.329
**CRP (mg/L)**				
Pre-LD	10.99 (1.97–24.14)	10.23 (1.54–19.13)	12.16 (4.44–61.21)	0.309
Peak	51.36 (15.12–101.20)	62.32 (16.54–103.99)	63.28 (30.81–118.18)	0.493
**IL-2 (pg/ml)**				
Pre-LD	2.06 (1.59–2.68)	2.11 (1.67–2.61)	1.81 (1.41–4.12)	0.670
Peak	3.03 (2.52–4.49)	2.76 (2.31–4.62)	3.50 (2.81–5.12)	**0.047**
**IL-4 (pg/ml)**				
Pre-LD	1.71 (1.45–2.99)	1.69 (1.41–2.83)	1.79 (1.53–3.12)	0.585
Peak	2.55 (2.23–2.97)	2.39 (2.21–2.99)	2.72 (2.24–3.55)	0.199
**IL-6 (pg/ml)**				
Pre-LD	9.33 (5.20–23.37)	6.37 (4.52–12.00)	18.09 (8.39–28.25)	**0.025**
Peak	50.74 (21.35–740.29)	38.35 (15.78–321.35)	55.23 (33.58–1244.67)	0.082
**IL-10 (pg/ml)**				
Pre-LD	4.66 (3.36–10.68)	3.71 (3.08–5.44)	9.13 (4.56–17.09)	**0.006**
Peak	23.86 (12.31–101.01)	20.80 (11.17–70.01)	81.36 (16.86–147.45)	**0.017**
**IFNγ (pg/ml)**				
Pre-LD	1.89 (1.57–2.80)	1.73 (1.57–2.63)	2.62 (1.45–3.05)	0.403
Peak	4.47 (2.88–11.00)	4.51 (2.90–13.27)	5.94 (3.24–16.04)	0.363
**TNFα (pg/ml)**				
Pre-LD	1.56 (1.25–2.88)	1.54 (1.26–2.45)	2.01 (0.92–3.04)	0.946
Peak	2.76 (2.16–4.08)	2.67 (2.03–3.77)	2.72 (2.18–3.41)	0.688

PLT indicates platelet; Pre-LD, pre-lymphodepletion; APTT, activated partial thromboplastin time;

FIB, fibrinogen; FDP, fibrinogen degradation products; TT, thrombin time; ATIII, antithrombin III; PT, prothrombin time; LDH, dehydrogenase; CRP, C-reactive protein; IL, interleukin; IFNγ, interferon γ; TNFα, tumor necrosis factor α.

Supplemental Table 8. Correlation matrix showing the correlation among coagulation markers, inflammation markers and tumor burden markers

	**D-dimer**	**PLT**	**FIB**	**PT**	**APTT**	**ATIII**	**TT**	**FDP**	**CRP**	**Ferritin**	**LDH**	**Bone-marrow blast**	**IL-6**	**IL-10**
**D-dimer**	1.0	-0.676, P<0.001	-0.519, P<0.001	0.664, P<0.001	0.473, P<0.001	0.170, P=0.244	0.508, P<0.001	0.924, P<0.001	0.360, P=0.007	0.669, P<0.001	0.745, P<0.001	0.488, P=0.057	0.609, P<0.001	0.568, P<0.001
**PLT**	-0.676, P<0.001	1.0	0.495, P<0.001	-0.536 P<0.001	-0.412, P=0.002	-0.108, P=0.462	-0.455, P<0.001	-0.696, p<0.001	-0.269, P=0.045	-0.662, P<0.001	-0.722, P<0.001	-0.734, P=0.002	-0.412, P=0.002	-0.558, P<0.001
**FIB**	-0.519, P<0.001	0.495, P<0.001	1.0	-0.502, P<0.001	-0.360, P=0.006	-0.037, P=0.801	-0.596, P<0.001	-0.516, P<0.001	0.032, P=0.813	-0.399, P=0.003	-0.545, P<0.001	-0.250, P=0.349	-0.340, P=0.010	-0.558, P<0.001
**PT**	0.664, P<0.001	-0.536 P<0.001	-0.502, P<0.001	1.0	0.644, P<0.001	-0.026, P=0.857	0.478, P<0.001	0.598, P<0.001	0.295, P=0.027	0.548, P<0.001	0.622, P<0.001	0.631, P=0.010	0.569, P<0.001	0.595, P<0.001
**APTT**	0.473, P<0.001	-0.412, P=0.002	-0.360, P=0.006	0.644, P<0.001	1.0	-0.075, P=0.607	0.456, P<0.001	0.532, P<0.001	0.539, P<0.001	0.582, P<0.001	0.450, P<0.001	0.174, P=0.519	0.578, P<0.001	0.497, P<0.001
**ATIII**	0.170, P=0.244	-0.108, P=0.462	-0.037, P=0.801	-0.026, P=0.857	-0.075, P=0.607	1.0	0.141, P=0.334	0.157 P=0.2831	0.045 P=0.757	0.168 P=0.253	0.154 P=0.291	0.515, P=0.044	-0.099 P=0.499	0.155 P=0.287
**TT**	0.508, P<0.001	-0.455, P<0.001	-0.596, P<0.001	0.478, P<0.001	0.456, P<0.001	0.141, P=0.334	1.0	0.495 P<0.001	0.105 P=0.441	0.501 P<0.001	0.427 P=0.001	0.474, P=0.065	0.479 P<0.001	0.315 P=0.018
**FDP**	0.924, P<0.001	-0.696, p<0.001	-0.516, P<0.001	0.598, P<0.001	0.532, P<0.001	0.157 P=0.283	0.495 P<0.001	1.0	0.395 P=0.004	0.696 P<0.001	0.744 P<0.001	0.447, P=0.084	0.728 P<0.001	0.628 P<0.001
**CRP**	0.360, P=0.007	-0.269, P=0.045	0.032, P=0.813	0.295, P=0.027	0.539, P<0.001	0.045 P=0.757	0.105 P=0.441	0.395 P=0.004	1.0	0.442 P=0.001	0.355 P=0.007	-0.065, P=0.814	0.448 P=0.001	0.456 P<0.001
**Ferritin**	0.669, P<0.001	-0.662, P<0.001	-0.399, P=0.003	0.548, P<0.001	0.582, P<0.001	0.168 P=0.253	0.501 P<0.001	0.696 P<0.001	0.442 P=0.001	1.0	0.632 P<0.001	0.688, P=0.004	0.429 P=0.001	0.611 P<0.001
**LDH**	0.745, P<0.001	-0.722, P<0.001	-0.545, P<0.001	0.622, P<0.001	0.450, P<0.001	0.154 P=0.291	0.427 P=0.001	0.744 P<0.001	0.355 P=0.007	0.632 P<0.001	1.0	0.494, P=0.005	0.510 P<0.001	0.585 P<0.001
**Bone-marrow blast**	0.4882, P=0.057	-0.734, P=0.002	-0.250, P=0.349	0.631, P=0.010	0.174, P=0.519	0.515, P=0.044	0.474, P=0.065	0.447, P=0.084	-0.065, P=0.814	0.688, P=0.004	0.494, P=0.005	1.0	0.274, P=0.054	0.679, P=0.304
**IL-6**	0.609, P<0.001	-0.412, P=0.002	-0.340, P=0.010	0.569, P<0.001	0.578, P<0.001	-0.099 P=0.499	0.479 P<0.001	0.728 P<0.001	0.448 P=0.001	0.429 P=0.001	0.510 P<0.001	0.274, P=0.054	1.0	0.483 P<0.001
**IL-10**	0.568, P<0.001	-0.558, P<0.001	-0.321, P=0.016	0.595, P<0.001	0.497, P<0.001	0.155 P=0.287	0.315 P=0.018	0.628 P<0.001	0.456 P<0.001	0.611 P<0.001	0.585 P<0.001	0.679, P=0.304	0.483 P<0.001	1.0

PLT indicates platelet; FIB, fibrinogen; PT, prothrombin time; APTT, activated partial thromboplastin time; ATIII, antithrombin III; TT, thrombin time; FDP, fibrinogen degradation products; CRP, C-reactive protein; LDH, dehydrogenase; IL, interleukin.

Supplemental Table 9. Risk stratification according to IL-10 level and the EASIX score on bleeding events

Risk group	Parameters		n*	Events, n	CumuInc, %	HR	95% CI	*P* value
	IL-10 (pg/mL)	EASIX						
**High risk**	>7.98	> 7.649	7	7	100.00	14.47	2.78–75.29	**< 0.0001**
**Intermediate risk**	>7.98	≤ 7.649	13	5	38.46	3.68	0.82 –16.67	0.089
	≤7.98	> 7.649						
**Low risk**	≤7.98	≤ 7.649	17	2	11.76	1 (reference)	-	-

IL indicates interleukin; EASIX, endothelial activation and stress index; CumuInc, cumulative incidence; HR, hazard ratio; CI, confidence interval.

High risk vs intermediate risk: HR, 5.495; 95% CI, 1.346–22.43; *P* = 0.0003.

*Only patients with available IL-10 levels were eligible for this analysis.

Supplemental Table 10. Univariate and multivariate logistic regression analysis of grade 2–3 bleeding events

Marker	Subgroup	No. case	No. Non-case	Unadjusted OR (95% CI)	P value	Adjusted OR (95% CI)	P value
**Pre-LD D-dimer (mg/L)**	≤0.58	0	21	1 (reference)	0.998		
	> 0.58	6	20	484642457.48 (0–)			
**Pre-LD CRP (mg/L)**	≤10.39	1	24	1 (reference)	0.101		
	>10.39	6	23	6.26 (0.70–56.10)			
**Pre-LD IL-6 (pg/ml)**	≤7.49	0	16	1 (reference)	0.998		
	>7.49	7	13	869871062.59 (0–)			
**Pre-LD IL-10 (pg/ml)**	≤7.98	0	24	1 (reference)	0.998		
	>7.98	6	5	1938569843.51 (0–)			
**Pre-LD Ferritin (ng/ml)**	≥96.3	4	32	1 (reference)	0.038		
	<96.3	2	1	16.00 (1.17–218.86)			
**Pre-LD EASIX**	≤8.81	2	41	1 (reference)	**0.002**	1 (reference)	**0.002**
	>8.81	4	3	27.33 (3.47–215.07)		27.33 (3.47–215.07)	
**Pre-LD PLT (10^9 /L)**	≥102.5	1	30	1 (reference)	**0.045**		
	<102.5	6	19	9.47 (1.06–84.96)			
**Pre-LD TNFα (pg/ml)**	≤2.93	3	24	1 (reference)	0.100		
	>2.93	3	5	4.80 (0.74–31.08)			
**CRS**	≤0.5	2	24	1 (reference)	0.301		
	>0.5	5	24	2.50 (0.44–14.17)			

OR, odds ratio; LD, lymphodepletion; CRP, C-reactive protein; IL, interleukin; EASIX, endothelial activation and stress index; PLT, platelet; TNFα, tumor necrosis factor α; CRS, cytokine release syndrome.

Supplemental Figure 1.

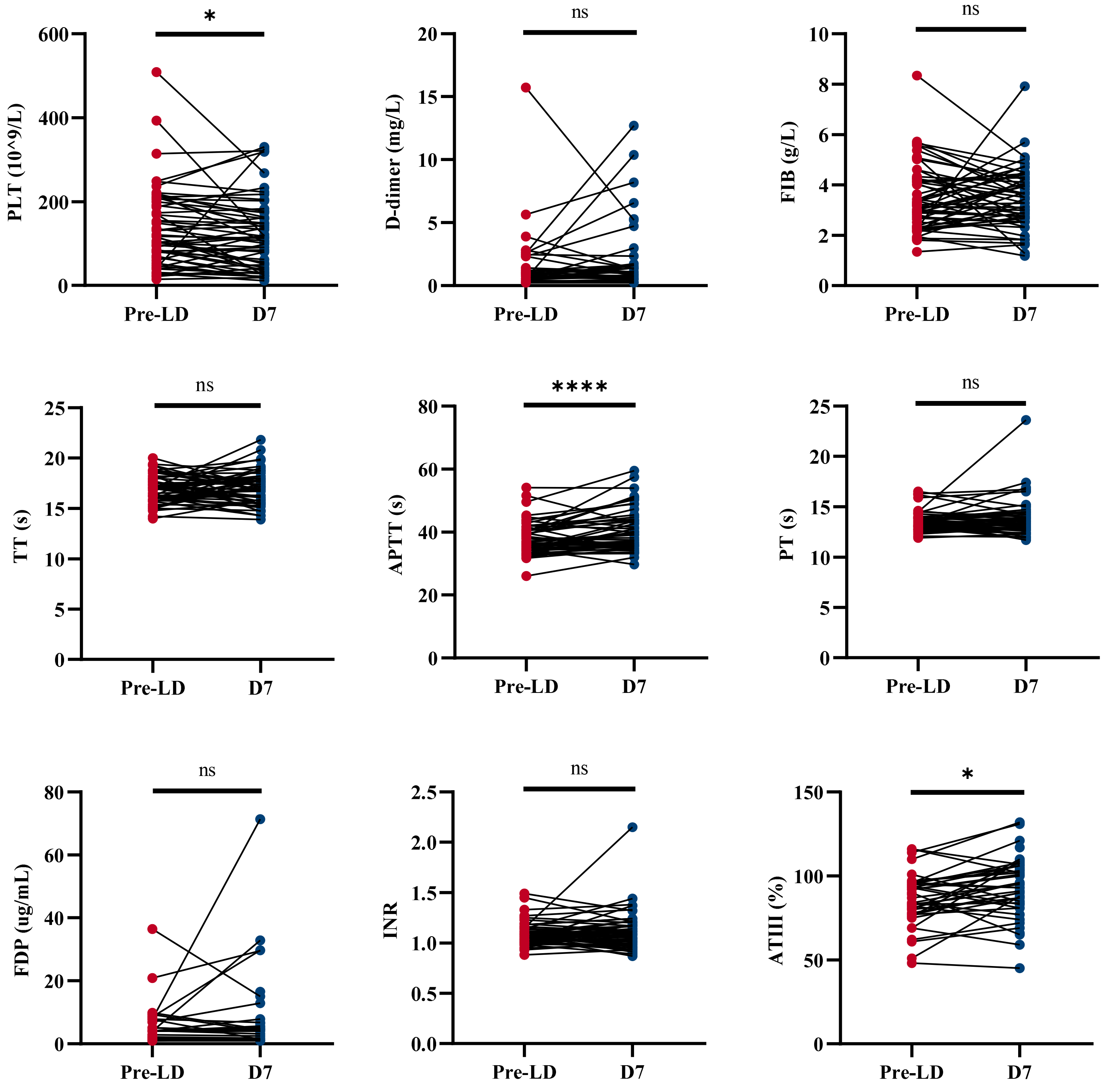

Supplemental Figure 1. Coagulation parameters changes from pre-LD to day 7. Changes of PLT, D-dimer, FIB, TT, APTT, PT, FDP, INR, and ATIII from pre-LD to day 7. **P* < .05. *****P* < .0001. ns, not significant. Pre-LD, pre-lymphodepletion; PLT, platelet; FIB, fibrinogen; TT, thrombin time; APTT, activated partial thromboplastin time; PT, prothrombin time; FDP, fibrinogen degradation products; INR, international normalized ratio; ATIII, antithrombin III.

Supplemental Figure 2.

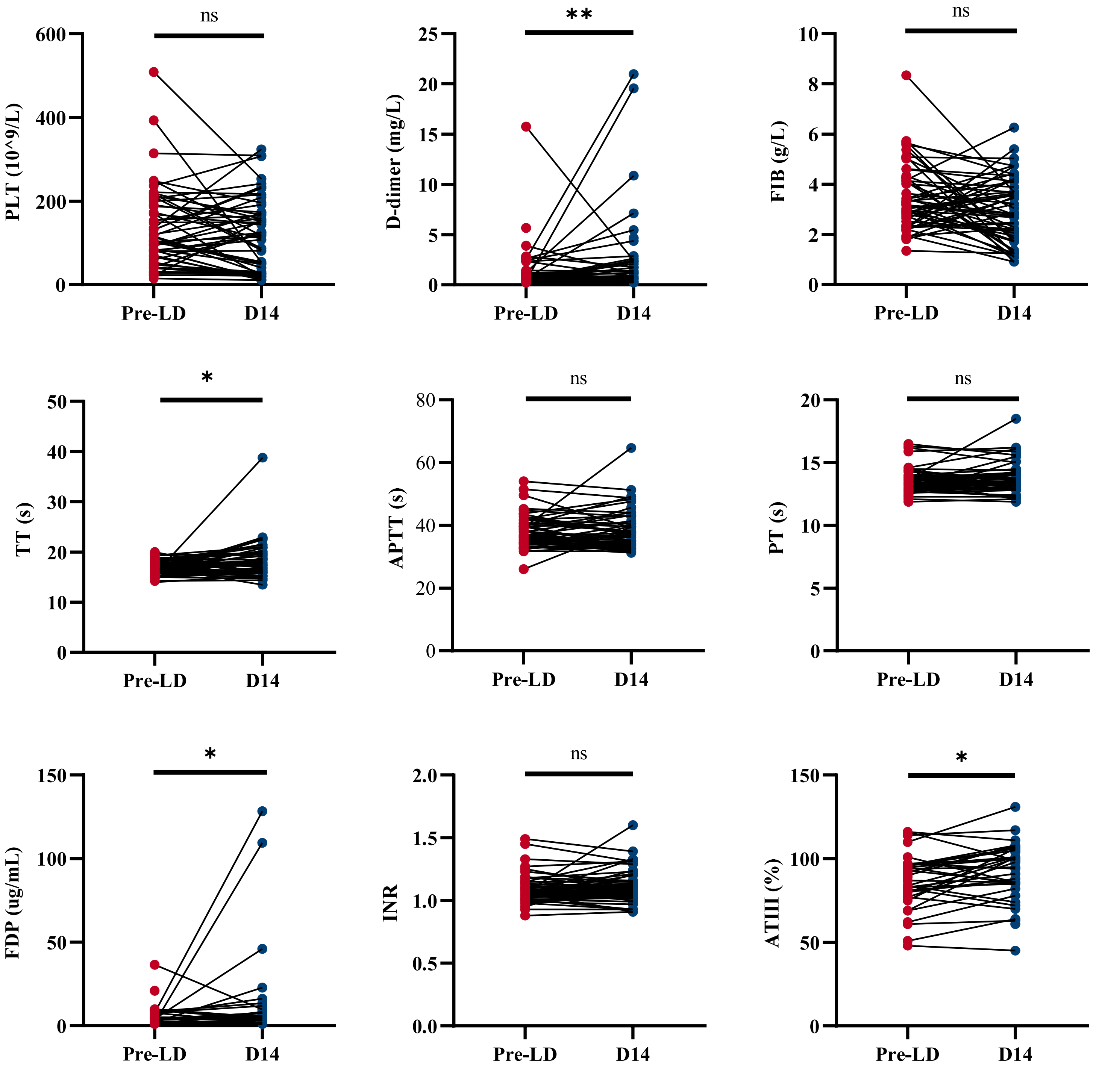

Supplemental Figure 2. Coagulation parameters changes from pre-LD to day 14. Changes of PLT, D-dimer, FIB, TT, APTT, PT, FDP, INR, and ATIII from pre-LD to day 14. *P < .05. **P < .01. ns, not significant. Pre-LD, pre-lymphodepletion; PLT, platelet; FIB, fibrinogen; TT, thrombin time; APTT, activated partial thromboplastin time; PT, prothrombin time; FDP, fibrinogen degradation products; INR, international normalized ratio; ATIII, antithrombin III.

Supplemental Figure 3.

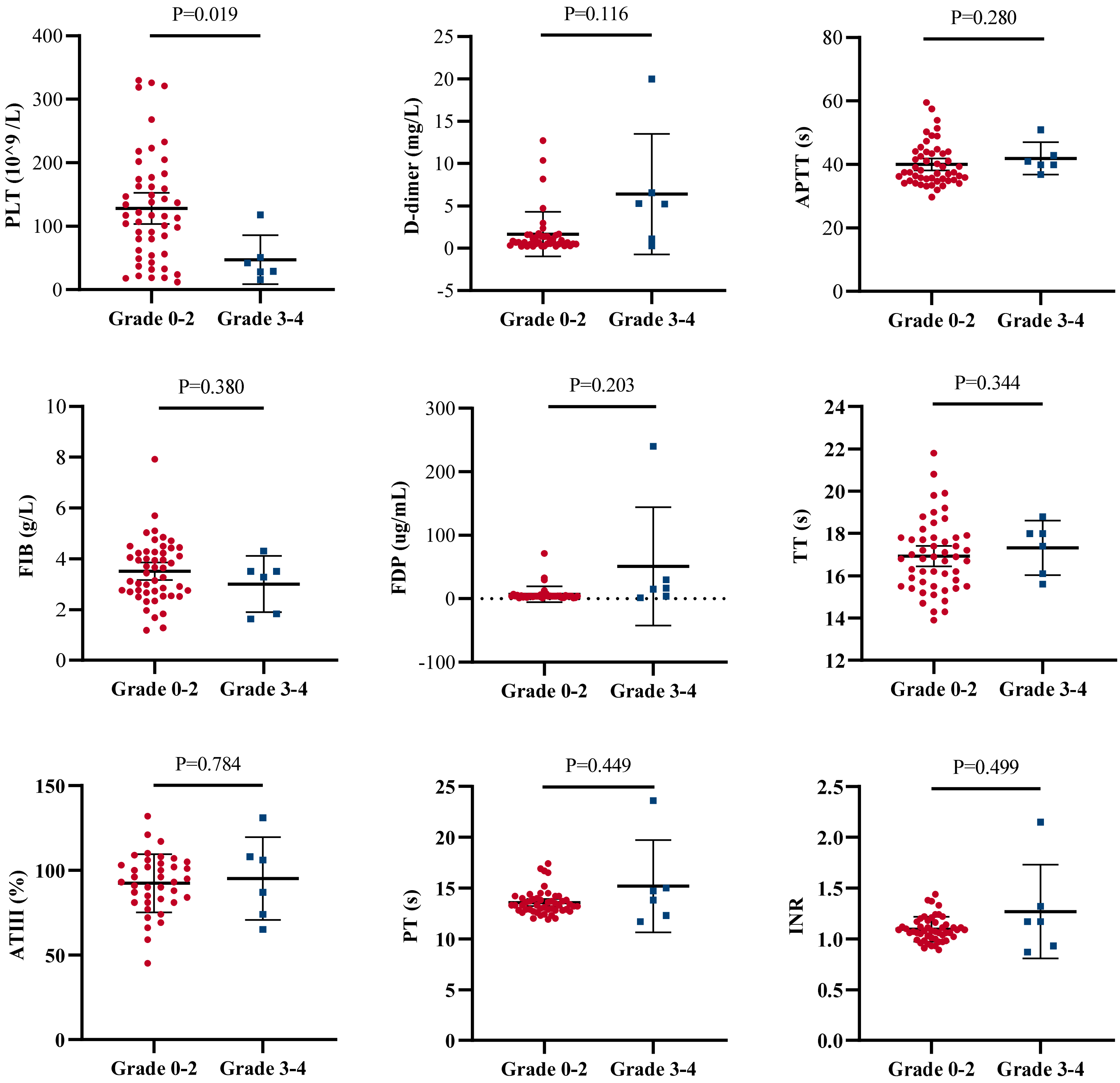

Supplemental Figure 3. Coagulation parameters by the severity of CRS on day 7. PLT, platelet; FIB, fibrinogen; TT, thrombin time; APTT, activated partial thromboplastin time; PT, prothrombin time; FDP, fibrinogen degradation products; INR, international normalized ratio; ATIII, antithrombin III.

Supplemental Figure 4.

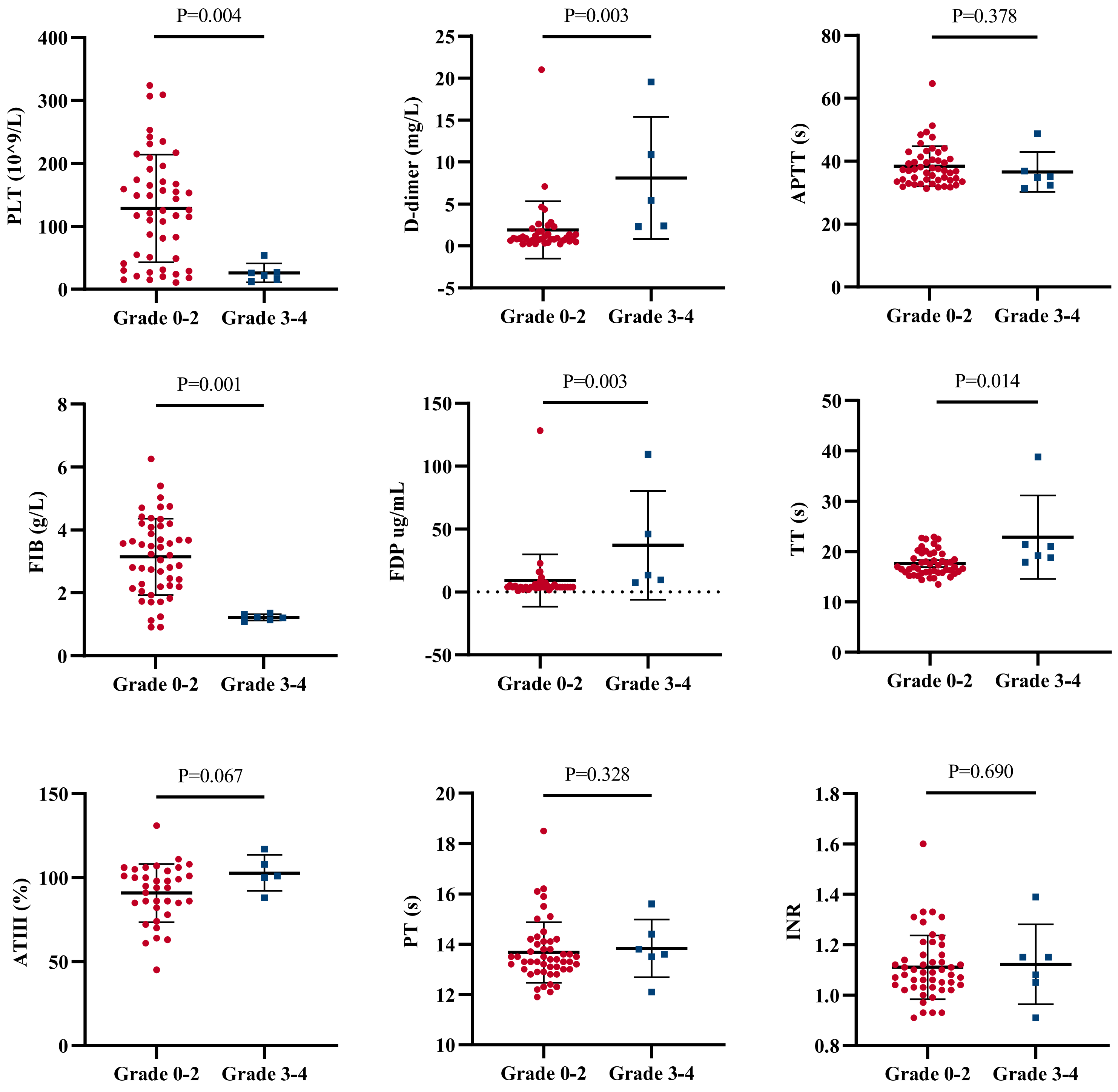

Supplemental Figure 4. Coagulation parameters by the severity of CRS on day 14. PLT, platelet; FIB, fibrinogen; TT, thrombin time; APTT, activated partial thromboplastin time; PT, prothrombin time; FDP, fibrinogen degradation products; INR, international normalized ratio; ATIII, antithrombin III.

Supplemental Figure 5.

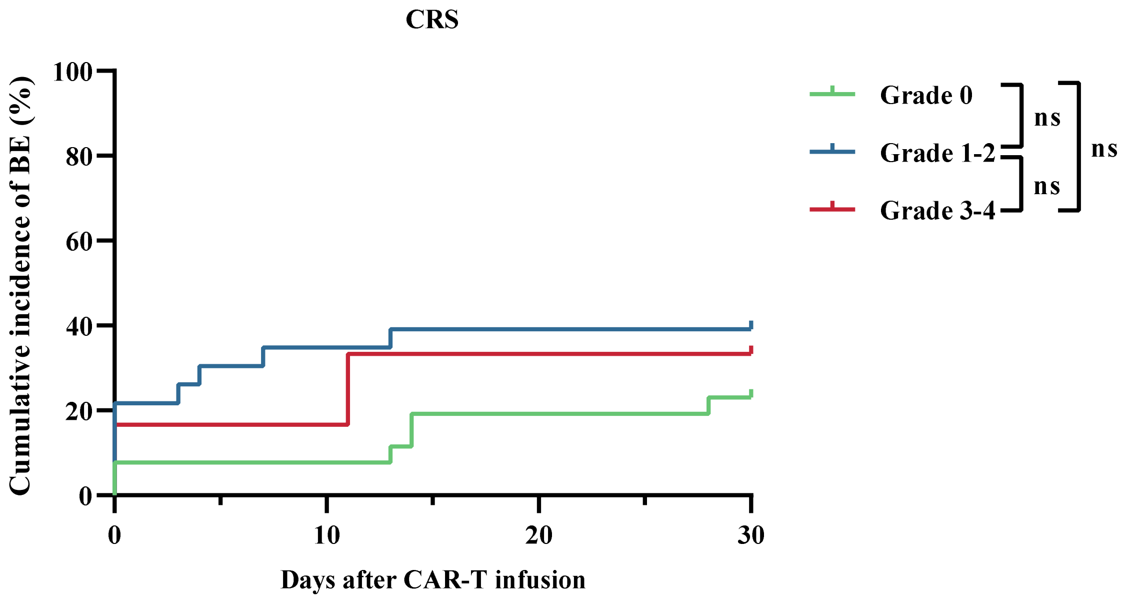

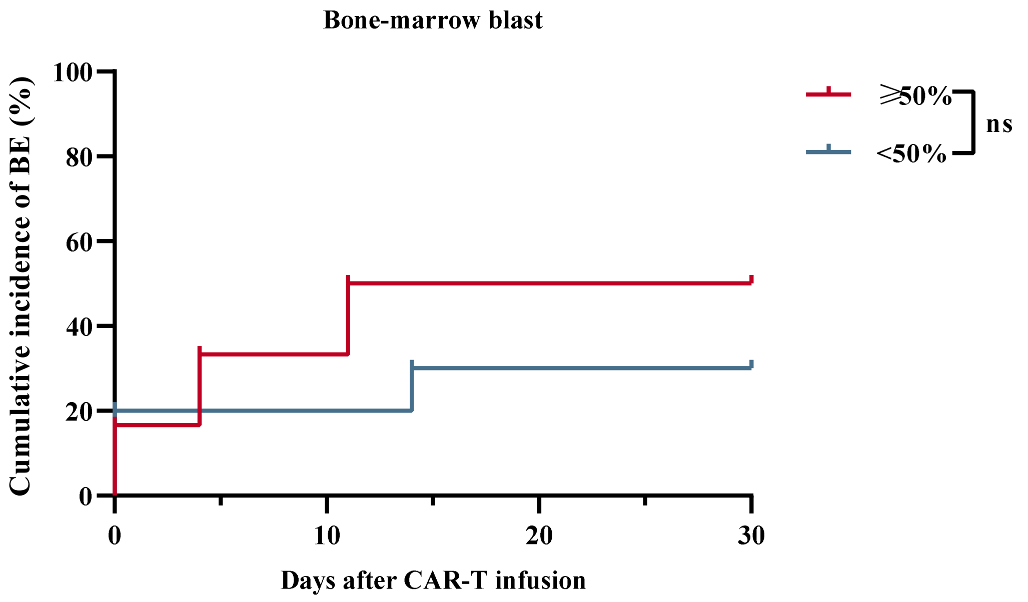

Supplemental Figure 5. Cumulative incidence of bleeding events by grade of CRS and bone-marrow tumor burden. BE, bleeding events; CRS, cytokine release syndrome.

Supplemental Figure 6.

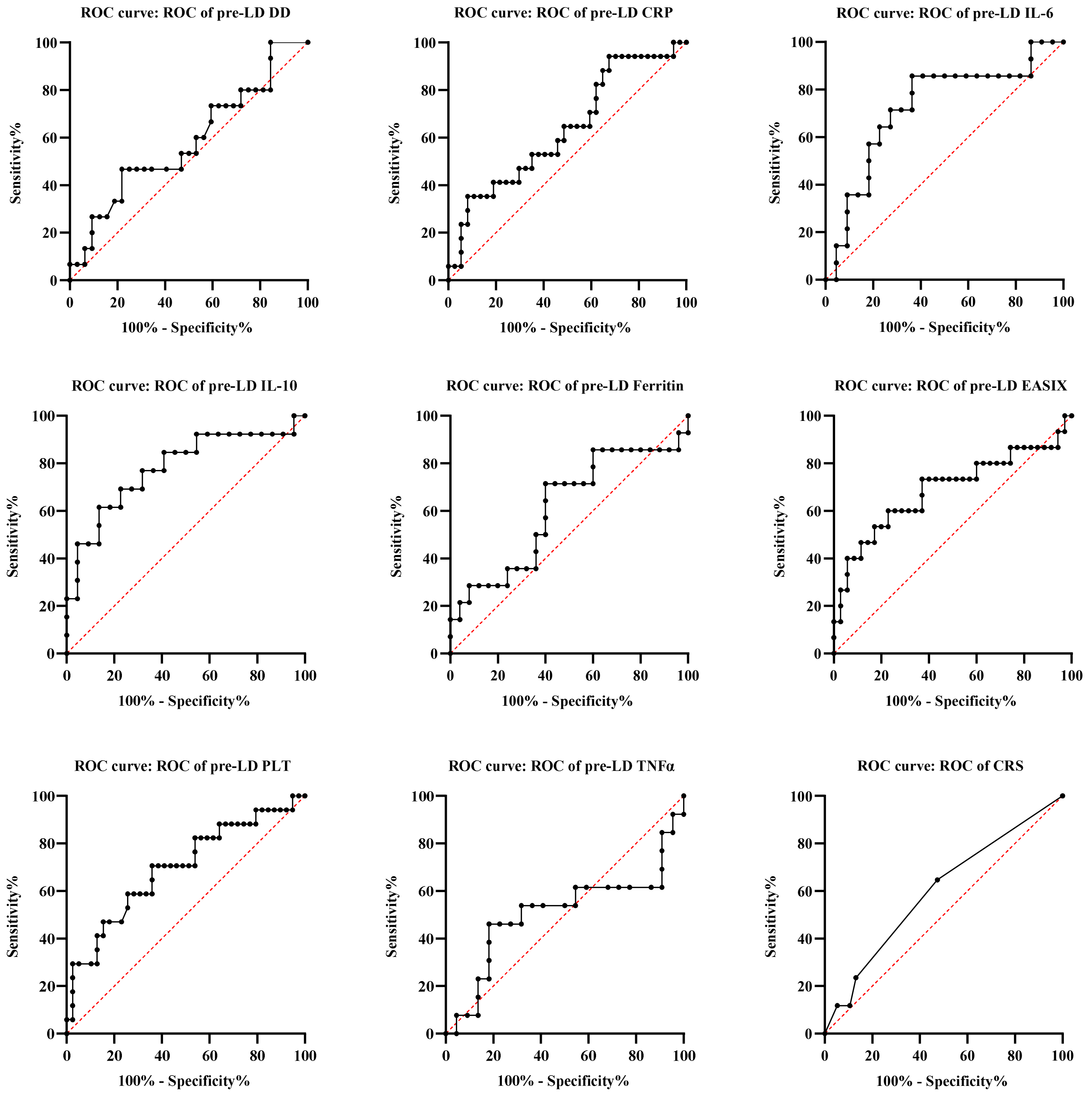

Supplemental Figure 6. ROC curve analysis of D-dimer, CRP, IL-6, IL-10, ferritin, EASIX score, PLT, TNFα, and CRS pre-lymphodepletion for bleeding events. ROC, receiver operating characteristic; CRP, C-reactive protein; IL, interleukin; EASIX, endothelial activation and stress index; PLT, platelet; TNF, tumor necrosis factor; CRS, cytokine release syndrome.

Supplemental Figure 7.

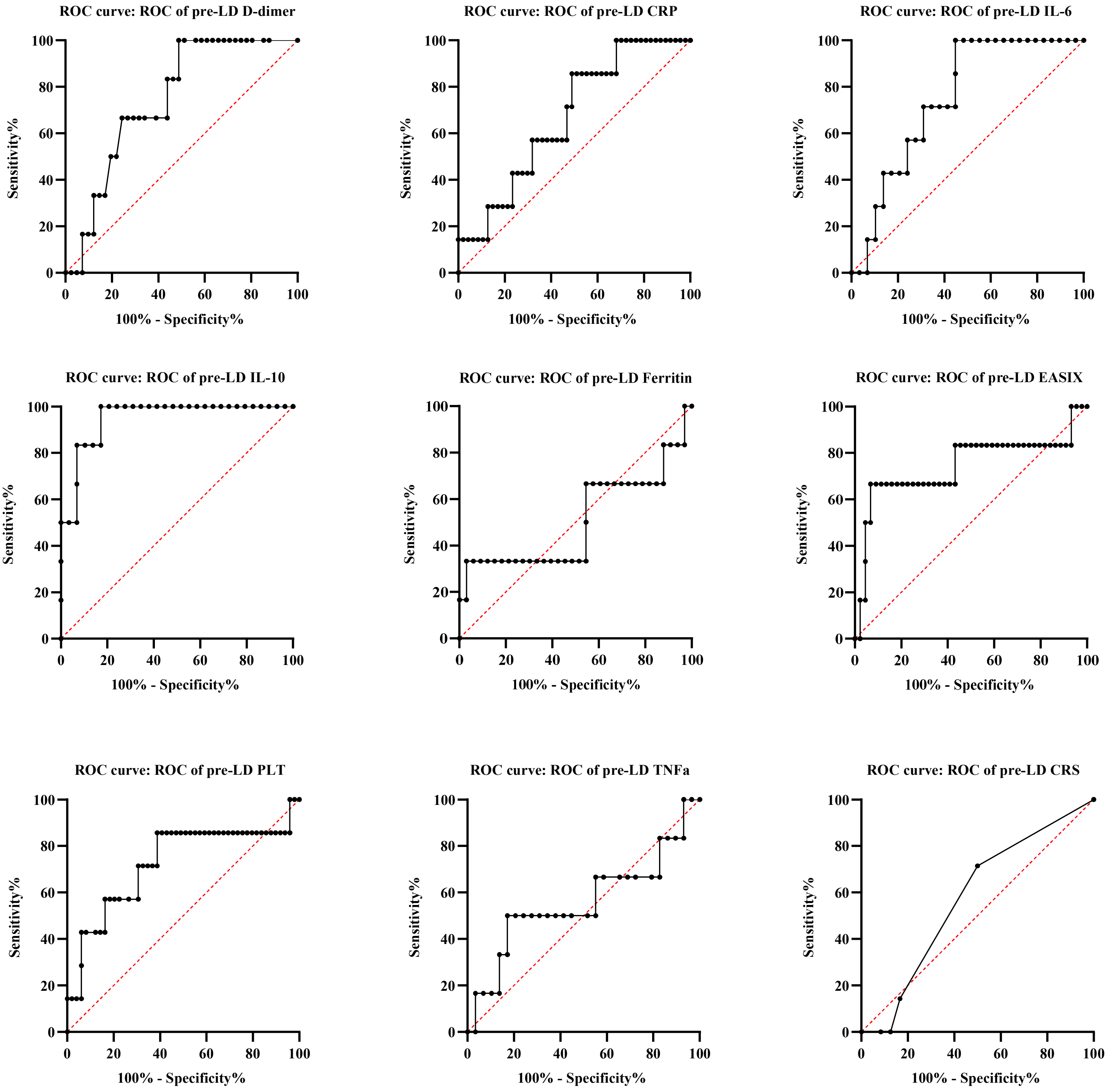

Supplemental Figure 7. ROC curve analysis of D-dimer, CRP, IL-6, IL-10, ferritin, EASIX score, PLT, TNFα, and CRS pre-lymphodepletion for grade 2-3 bleeding events. ROC, receiver operating characteristic; CRP, C-reactive protein; IL, interleukin; EASIX, endothelial activation and stress index; PLT, platelet; TNF, tumor necrosis factor; CRS, cytokine release syndrome.
